# Simplified Formulae for the Estimation of Offshore Wind Turbines Clutter on Marine Radars

**DOI:** 10.1155/2014/982508

**Published:** 2014-03-17

**Authors:** Olatz Grande, Josune Cañizo, Itziar Angulo, David Jenn, Laith R. Danoon, David Guerra, David de la Vega

**Affiliations:** ^1^Department of Communications Engineering, University of the Basque Country (UPV/EHU), Alda. Urquijo s/n, 48013 Bilbao, Spain; ^2^Department of Electrical & Computer Engineering, Naval Postgraduate School, Monterey, CA 93943, USA; ^3^School of Electrical and Electronic Engineering, The University of Manchester, Sackville Street Building, Manchester M13 9PL, UK

## Abstract

The potential impact that offshore wind farms may cause on nearby marine radars should be considered before the wind farm is installed. Strong radar echoes from the turbines may degrade radars' detection capability in the area around the wind farm. Although conventional computational methods provide accurate results of scattering by wind turbines, they are not directly implementable in software tools that can be used to conduct the impact studies. This paper proposes a simple model to assess the clutter that wind turbines may generate on marine radars. This method can be easily implemented in the system modeling software tools for the impact analysis of a wind farm in a real scenario.

## 1. Introduction

Offshore wind energy sector has grown strongly in the last decade, and greater turbine sizes are being developed [1–6]. Offshore wind farms are sometimes deployed near radar sites, and they may cause clutter degradation in shore-based VTS (vessel traffic service) radars and ship-borne radars, because the severe echoes they generate reduce the detection and measurement capabilities of the radar in the area around the wind farm [7–10]. In some specific cases, the rotation of the blades may generate tracks initiation in VTS radars that use tracking algorithms, due to fluctuation of turbine returns. These effects have led to the research of some mitigation techniques [11–14].

The potential degradation in the proper radar operation may have an impact on security, and the potential impact should be analyzed before the wind farm is installed. When assessing the impact of a particular wind farm, the accuracy of the assessment on these radars is highly dependent on the scattering models used to calculate the scattered signals from wind turbines.

The term radar cross section (RCS) refers to the amount of scattered power from a target towards the radar, when the target is illuminated by the radar signal [15]. Although some authors propose that the parameter RCS cannot be applied to wind turbines (and to any object on the ground) because the plane wave condition is not entirely fulfilled [16], the main authorities in the area of telecommunications and radar that have published guidelines for analyzing the impact of wind turbines use this parameter to account for the scattering of wind turbines and evaluate the potential impact a wind farm may cause [7, 17–23]. Due to this fact, the model proposed in this paper is based on the concept of the radar cross section of complex objects. The calculation of the RCS of a wind turbine is a complex process that involves the combination of scattered signals generated from components made of different materials, some of them continuously time-varying in their orientation and rotation speed depending on the wind conditions.

Although the conventional methods for RCS estimation provide accurate values for a specific rotor orientation and a particular position of the blades, these values are not directly applicable to system modeling algorithms that aim to assess the impact of a turbine in different working scenarios, as the RCS values significantly vary with radar aspect angle and blade position. Furthermore, all of the conventional methods require extremely detailed representations of the turbine, which require significant modeling and computational effort in generating a computer model with the shape and dimensions of the components of the wind turbine. Additionally, a different calculation must be made for each blade position and rotor orientation. As a consequence, the conventional models are not easily implemented in computer simulation tools for analyzing the potential impact of a specific wind farm on surrounding marine radars.

In the analysis of the potential impact of wind turbines on radiocommunication services, the regulatory bodies propose the use of representative RCS values [17–23], in order to use simple models and avoid the complexity that the statistical characterization of the wind turbine RCS would lead to. Accordingly, the aim of this paper is to provide a method for obtaining representative RCS values of the whole wind turbine, independently of the blades position, which can be easily included in the guidelines published by these regulatory bodies.

Therefore, it is desirable to have a simplified analysis procedure that can be directly implemented in a software tool for estimating the impact of real wind farms [24, 25]. Other approaches have been described in the literature for estimating the level of different clutter sources without needing the use of sophisticated models. For example, rain and sea clutter estimations have been made by simple models based on sea state and significant wave heights parameters [26] or by statistical models with a Hilbert-based predetection stage [27]; sea clutter estimations have been made by artificial neural network-based models [28], or the suite of techniques presented in [29] for wind turbine clutter mitigation. From the analysis of these and other techniques, it can be concluded that the procedure must provide the variation of the RCS with dimensions and materials of the wind turbine and the operational parameters of the marine radars (frequency, polarization, and antenna pattern) but must avoid the use of detailed wind turbine geometry. Additional conditions, such as the range between radar and turbine, as well as any important near field scattering effects, should be also included in the model.

## 2. Objective

The aim of this study is to develop a simple model for obtaining representative values of scattered signals from an offshore wind turbine, in order to evaluate the potential impact on marine radars. Hence, the purpose is not to obtain detailed RCS values for specific blade positions of a wind turbine but a first approximation of the clutter level received by the radar antenna, in order to determine whether a new wind farm may degrade the detection capability of the nearby marine radars. The method must be implementable in algorithms for the assessment of wind turbine impact in real scenarios.

Scattered signals from the turbine will be assessed by characterizing the RCS of the wind turbine, and, therefore, the clutter level received by the radar may be determined. Specific operating conditions of marine radars and offshore wind turbines will be applied to obtain an ad hoc method for this situation. Primarily, they are the frequency bands used by the marine radars [30] and the particular operating conditions of the wind turbines and radar, such as turbine dimensions, materials, incidence angles of the radar signal, and variability of the scattered signals due to the rotating blades and the rotor orientation.

The proposed method will allow impact studies of offshore wind farms to be easily conducted within the coverage area of marine radars. The results provided by this method will be helpful for wind farm promoters and administrations to determine the adequacy of the proposed wind farm.

## 3. Theoretical Basis and Main Considerations of the Analysis

The physical optics (PO) method is one of the most commonly used approximations for obtaining backscattered RCS values [31–34]. This high frequency method is applicable when the wavelength of the incident radiation is small with respect to the characteristic dimension of the body (*L* ≪ 10*λ*). The PO method approximates the current on the illuminated parts of the perfectly conducting surface by $2\hat{n}\times \vec{H_{i}}$, where $\vec{H_{i}}$ is the incident magnetic field intensity (A/m) and $\hat{n}$ is the normal to the surface. The current is assumed to be zero in the shadows. Generally, if the wind turbine is in the far field of the radar, the incident field is a plane wave; however, in the near field, it would be a spherical wave [15].

The methodology followed in this study is to start with the analysis of the scattering patterns of the major components that compose the wind turbine: mast, nacelle, and blades. Simplified expressions based on the PO approximation are developed for the mast of the turbine, taking into account the likely operating conditions of the offshore wind farms and marine radars. The complex shapes of the nacelle and blades preclude the use of simplified PO formulae for calculating their RCS values. For these components, complicated models and numerical solutions of Maxwell's equations must be used for accurate calculation of RCS values.

A discussion of applicable computational electromagnetic (CEM) techniques and their advantages and disadvantages is given in [34]. Generally, the software simulations require detailed surface discretization of the wind turbine models into facets. The allowable size of the facets depends on the simulation method and desired accuracy. For the simulation tool used here [33], the PO approximation is used to obtain the scattered field from each facet of the surface turbine model, and superposition is used to combine the scattered signals from all facets that compose the model to find its RCS [31, 33, 34].

**Figure 1:**
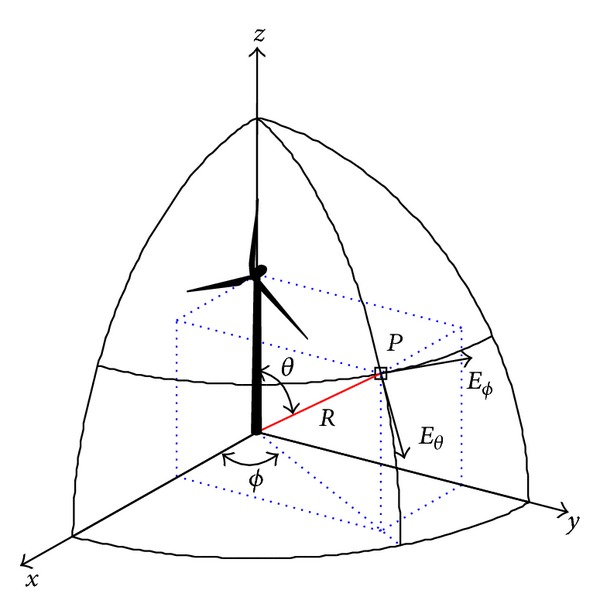
Spherical coordinate system used in the RCS calculations.

The specific conditions that are applicable to marine radars and offshore wind farms allow some simplification:

- although the scattering is a bistatic phenomenon with great dependency on the aspect angle, marine radars only receive monostatic backscattered signals; therefore, the analysis is adapted to monostatic backscattering values;
- ship-borne marine navigational radars are often located a few meters above the sea surface, while shore-based VTS radars are located at low elevations near the coastline; hence, the wave front from the radar propagates parallel or almost parallel to the sea surface (depending on atmospheric refraction); assuming that the ship pitch and roll is only a couple of degrees leads to incidence angles on the wind turbine nearly perpendicular to the vertical axis of the mast (*θ* = 90° in Figure 1) or just a few degrees above the sea surface; in this study, the range 87° < *θ* < 90° has been deemed as representative of this scenario;
- since the far field monostatic received power varies as 1/*R* ^4^, where *R* is the range between the radar and wind turbine, the disturbance of wind turbines is greater for radars located close (a few kilometers) to the wind farm; as most of the offshore wind farms are located between 2 km and 15 km from the coastline [35], far field condition of the scattering is not fulfilled in the cases of impact, and near field effects of the scattering must be considered; these effects are described in Section 7.

## 4. Calculation of RCS by means of Simulation Tools

### 4.1. Description of the Wind Turbine Models Used in the Simulation Tools

Any large, smooth surface can be approximated by a collection of small triangles placed edge-to-edge over the surface [31]. When discretizing the surface in preparation for applying the PO approximation, large triangles can be used, if the surface is flat. For curved surfaces the facet size must be fine enough (i.e., sufficiently small triangle edges) so that a “tight” fitting mesh can be generated. Even so, when a curved surface is approximated by a triangular mesh, facet noise will occur (it is also called facetization error) [34].

**Figure 2:**
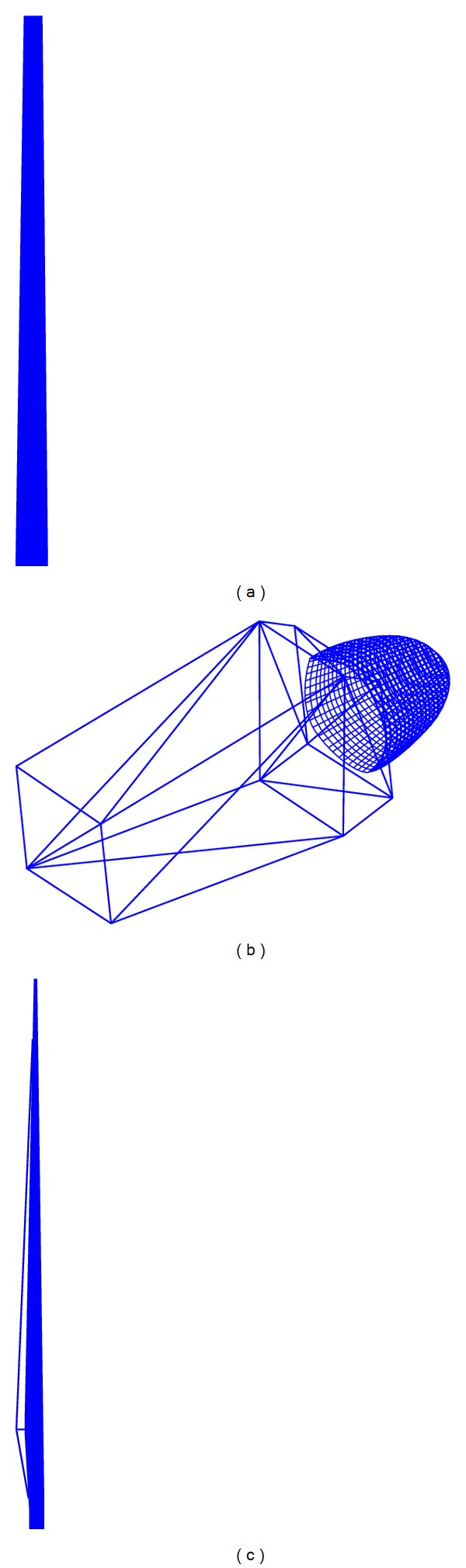
(a) Facets-based design of the mast. (b) Facets-based design of the nacelle. (c) Facets-based design of the blade.

**Figure 3:**
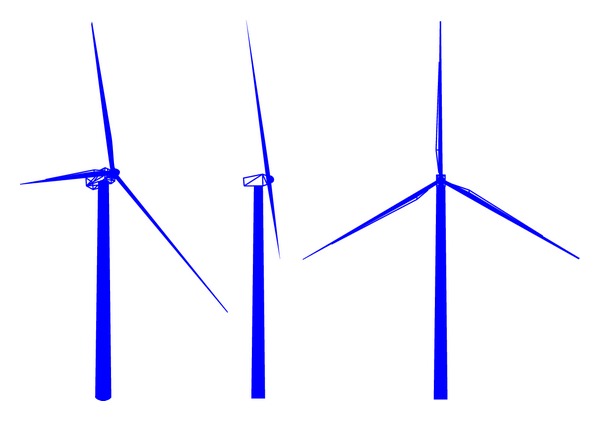
Facets-based representations of the whole wind turbine.

Three real offshore wind turbine models of different dimensions have been used in the RCS estimations (see Table 1 for dimensions). The facets-based wind turbine representations used in this study are shown in Figures 2 and 3. The mast is represented as a truncated cone with very slight slope, since this is the geometric shape that best represents the different offshore wind turbine tower models. Tower and blade bending due to wind loading is not considered in the model.

### 4.2. Description of the PO Software Tools

Two different simulation tools for RCS characterization were used. The first one is POfacets [33], which uses the PO approximation to predict the far field radar cross section patterns of complex targets meshed into triangular facets. Written in Matlab, POfacets uses the PO method to calculate the currents induced on each facet by the incident plane wave, and then the scattered field from each facet is computed by means of radiation integrals, and the results are summed to get the total RCS [31–34].

The second software tool (RePORA) was developed by the University of Manchester and is part of the wind farm impact modeling suit WinR. The RePORA model imports the simplified turbine geometry and segments the turbine's principal components into small sections. Each section is then meshed into quadrilateral facets and modified PO formulations are used to compute the near field monostatic scattering of each section. Each section is then assumed as a point scatterer positioned at the centre of the relevant section with phase referenced to this point. The total RCS can then be computed by coherently adding the contributions from all the turbine segments [36].

POfacets was used for obtaining wind turbine backscattering patterns in the far field, while RePORA was used for the analysis of near field effects in the scattering phenomenon.

Although the above-mentioned methods provide accurate RCS values for complex targets at high frequencies, both of them require specialized models of the wind turbine at each blade position and rotor orientation. For these reasons, the tools cannot be easily integrated in the algorithms of an impact study software tool. However, these tools will be used for the verification of the proposed simplified model against other PO-based models.

## 5. Characterization of the Backscattering Pattern of the Wind Turbine

Firstly, the scattering patterns for the whole wind turbine and for each of its elements are obtained and compared. Two representative radar frequencies have been used in the analysis: 3.08 GHz (S band) and 9.445 GHz (X band) [30].

**Figure 4:**
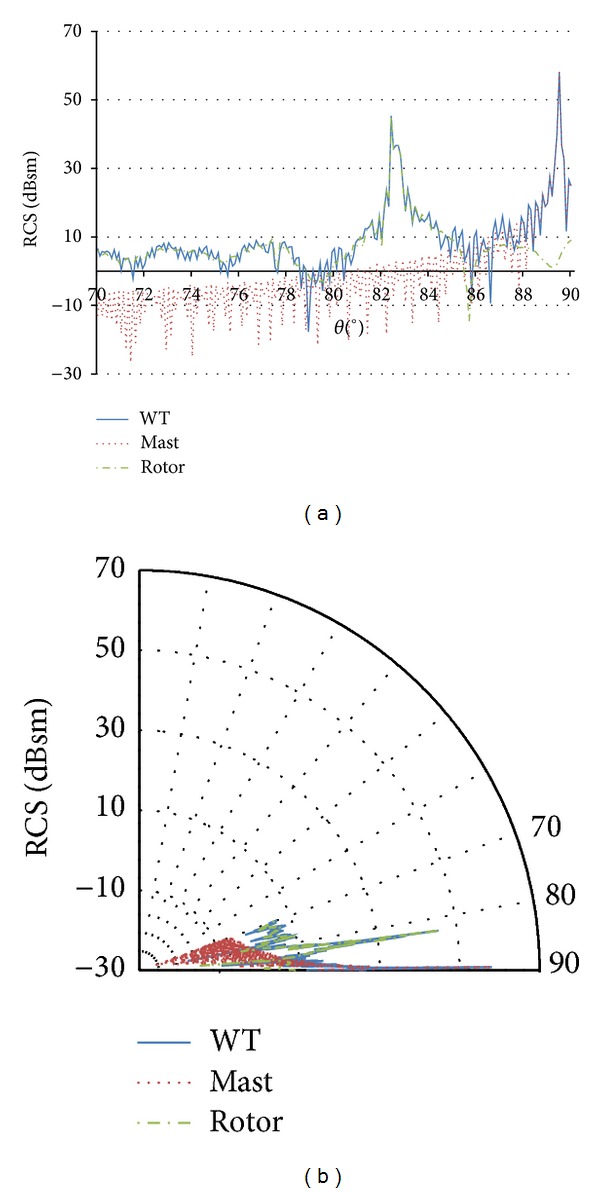
Wind turbine (blue line), mast (red dotted line), and rotor (green dotted line) RCS vertical pattern for the turbine model 1 at 3.08 GHz (*ϕ* = 30° and 70° < *θ* < 90°). Polar (a) and Cartesian (b) coordinate representations.

**Figure 5:**
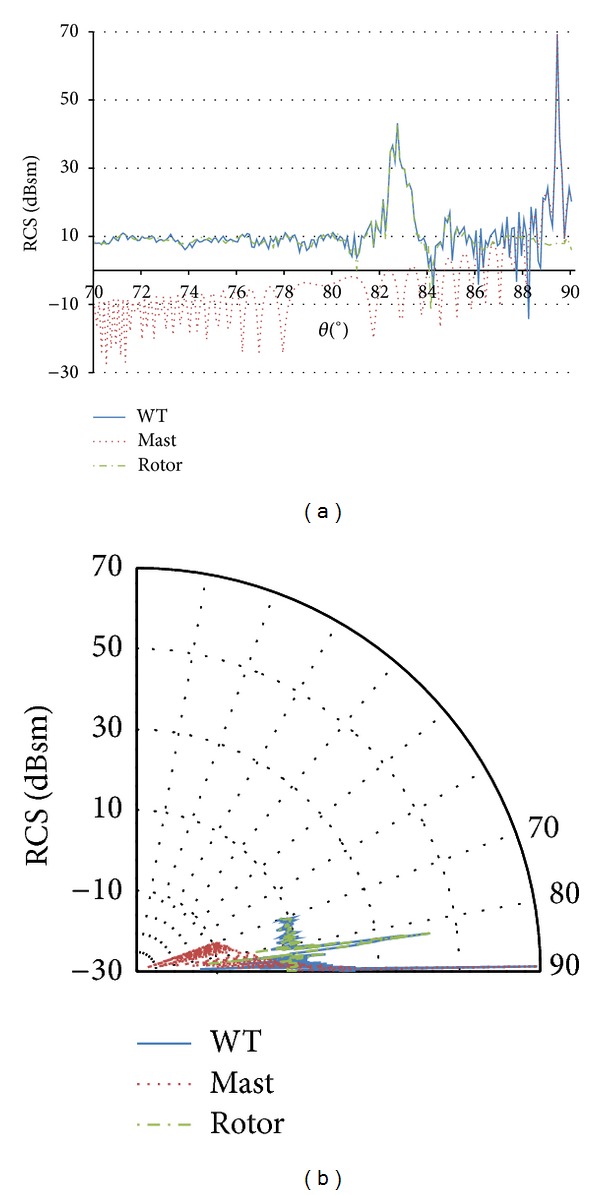
Wind turbine (blue line), mast (red dotted line), and rotor (green dotted line) RCS vertical pattern for the turbine model 3 at 9.945 GHz (*ϕ* = 30° and 70° < *θ* < 90°). Polar (a) and Cartesian (b) coordinate representations.

Due to the reduced tapering of the cylindrical mast (upper diameter is slightly smaller than lower diameter) and the relative location of marine radar antennas and offshore turbines, the incident angle on the mast is always within or close to the angle, where the maximum RCS value is obtained. Consequently, only incidence angles parallel, or almost parallel, to the sea surface (87° < *θ* < 90°) are of interest for the analysis of marine radars and offshore wind turbine scenario.  Figure 4 shows RCS vertical patterns of the mast, rotor, and the whole wind turbine for turbine model 1 at 3.08 GHz, and Figure 5 includes the corresponding results for the largest turbine model (model 3) at the highest frequency (9.445 GHz). In both cases, only the range 70° < *θ* < 90° is presented in the graphs.

For most of the incidence directions, results show significant variations of the RCS values with different rotor orientations and blade positions, because the RCS values of the mast are quite lower than the RCS values of the rotor, and, therefore, the RCS variation of the whole wind turbine follows the variation of the rotor. But, in the specific conditions of the scenario under analysis (monostatic radar and incidence direction of 87° < *θ* < 90°), results of the analysis show that backscattering from the mast is significantly higher than from the rotor and not dependent on blade rotation. Therefore, for these specific conditions, there is no remarkable variation in the RCS value of the whole turbine, and no statistical analysis is necessary for the RCS characterization. Similar results were obtained for other rotor orientations and blade positions. In addition, in the simulations, the blades are supposed to be metallic, while modern blades are made of composite materials, whose RCS is expected to be significantly lower. In conclusion, even assuming this worst case assumption with respect to the scattering from the rotor, the mast remains the main scatterer of the wind turbine for every blade position.

As it can be observed in the figures, the highest values of RCS in the angular range of interest for marine radars are 89.50°, 89.51°, and 89.43°, for turbine geometry models 1, 2, and 3, respectively. A comparative of the scattering caused by the mast and in different blades has been also carried out, considering the rotor orientation that causes maximum scattering from the blades. In all the cases, the mast is clearly the main contributor to the total RCS of the wind turbine. Moreover, the strong directivity in the vertical plane of the scattering pattern is because the mast height is much greater than the wavelength of the incident wave [15]. The high RCS values at other elevation angles are not of interest as stated in assumption 2 in Section 3.

**Figure 6:**
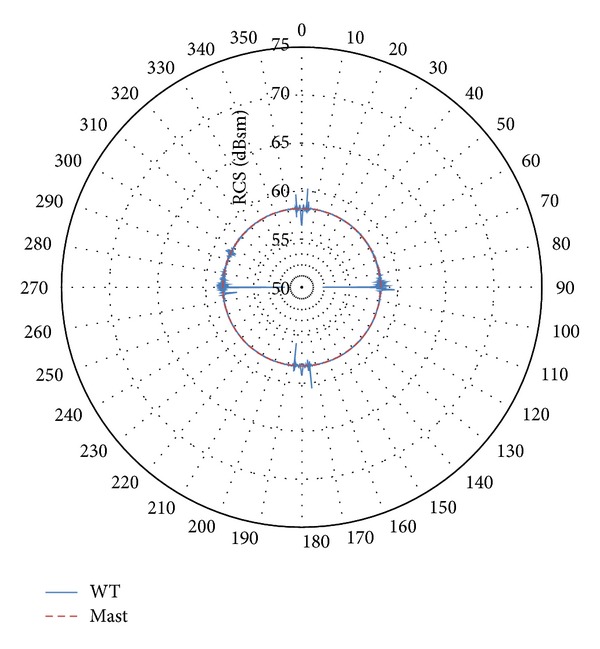
Variation in the horizontal plane of the RCS pattern for the maximum RCS value of the vertical pattern (incidence normal to the mast surface). Wind turbine (blue line) and mast (red dotted line) RCS patterns for turbine model 1 at 3.08 GHz.

**Figure 7:**
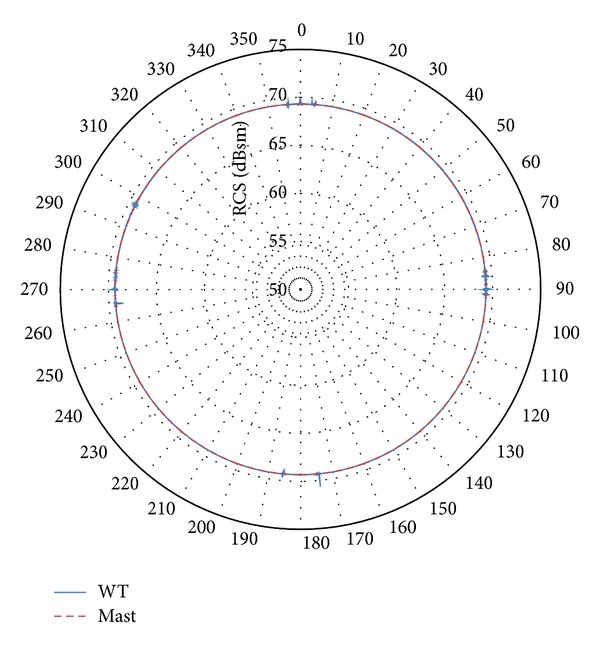
Variation in the horizontal plane of the RCS pattern for the maximum RCS value of the vertical pattern (incidence normal to the mast surface). Wind turbine (blue line) and mast (red dotted line) RCS patterns for turbine model 3 at 9.945 GHz.

Accordingly, it is reasonable to consider the mast as the main scatterer for the scenario of interest [37]. But, in addition, there is some variation in the scattering pattern as the rotor changes its orientation with respect to the radar (variation in *ϕ*). Figures 6 and 7 show the horizontal plane pattern through the maximum value in the vertical pattern (incidence normal to the surface of the mast). These figures represent the RCS as the rotor varies its orientation with respect to the radar location.

As it can be observed, the mast is the dominant contributor to RCS at all angles, regardless of the orientation of the rotor with respect to the radar location. For a few specific orientation occurrences, the rotor provides slightly higher values (1-2 dB); high values at 90° and 270° are caused by the side flat surfaces of the nacelle, and high values at other directions are caused by blades.

As a result, it is assumed that the mast provides a representative RCS value for all the directions of the rotor orientation, if the maximum RCS value in the vertical plane is selected. Accordingly, the mast may be considered as the primary scatterer for these incident angles, since the RCS from the mast equals or exceeds any scattered signal from other parts of the wind turbine [37]. Therefore, this paper will assume that the RCS of a turbine can be approximated by the RCS of the tower only. This will greatly reduce the computational complexity and the runtime required. It will also provide an easy-to-integrate methodology for wind farm assessment on marine radars. Although this approach does not account for the RCS variation due to the rotation of the blades, this is deemed acceptable for marine navigational radars and for providing initial assessment for the more complex VTS radars. Such radars commonly do not rely on Doppler processing; thus the movement of the blades may not be considered an issue.

## 6. Characterization of the Backscattering Pattern of the Mast

Considering that the mast is the main reflector for analyzing the impact of offshore wind farms on marine radars, the next step in this study is to obtain a simplified RCS model to be used in the algorithms for analyzing the impact on radar performance.

The PO approximation is the most convenient model for assessing the backscattered RCS of the mast, represented as a perfectly conducting tapered cylinder. A closed-form expression for the RCS due to a linearly polarized normal incident wave is [38]

**Figure 8:**
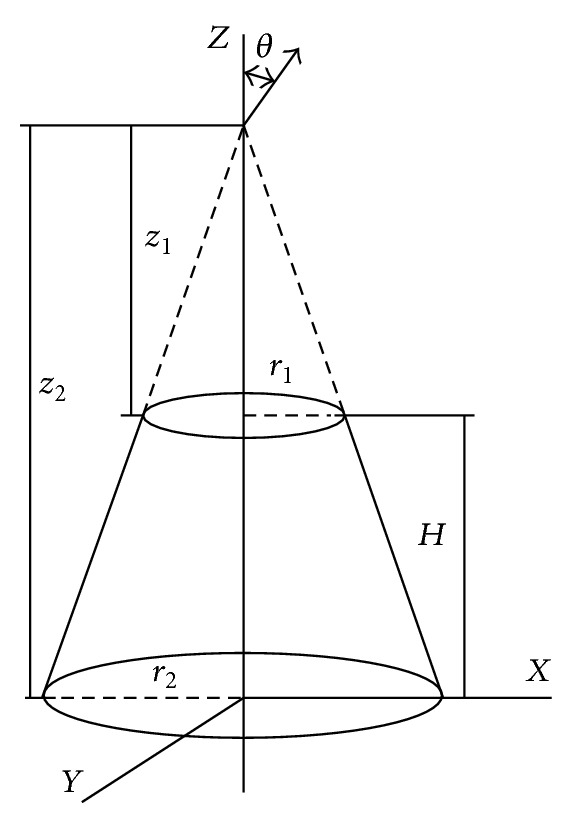
Geometry for the RCS calculation of a truncated cone (frustum) [38].

(1) σθn=8·π·(z23/2−z13/2)29·λ·sin⁡θn·tan⁡α ·(sin⁡θn−cos⁡⁡θn·tan⁡α)2 for  θ=θn,

where *λ* is the wavelength of the radar transmission, *θ*, *z*_1_, *y*, and *z*_2_ are defined in Figure 8, *θ*_*n*_ is the aspect angle at normal incidence, and *α* is the half cone angle defined as

(2) $$\begin{array}{c} \alpha =\arctan\left(\frac{r_{2}-r_{1}}{H}\right). \end{array}$$

Applying trigonometric identities to (1),

(3) $$\begin{array}{c} \sigma_{\theta_{n}}=\frac{8\cdot \pi \cdot {\left(z_{2}^{3/2}-z_{1}^{3/2}\right)}^{2}}{9\cdot \lambda }\cdot \frac{\sin\alpha }{{\left(\cos\alpha \right)}^{4}}. \end{array}$$

**Figure 9:**
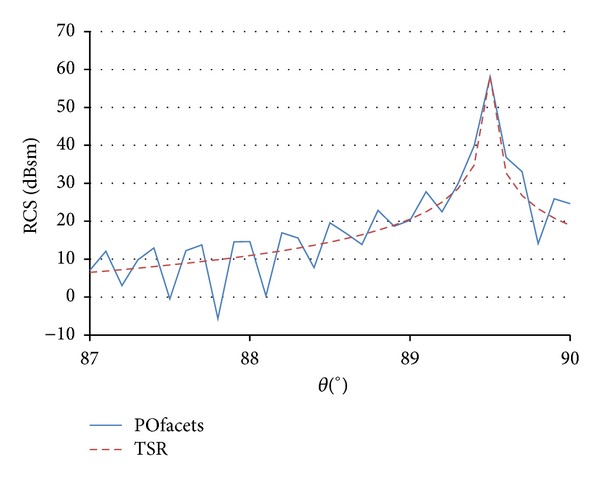
Comparison between the RCS values of the mast obtained from POfacets tool (blue line) and from the TSR simplified model (red dotted line) (turbine model 1 at 3.08 GHz).

The accuracy of this approximation must be assessed in order to evaluate its suitability for the case under study.  Figure 9 shows the comparison between the results of monostatic RCS calculation provided by (1) and the results provided by POfacets for turbine model 1 at 3.08 GHz. As it is shown in the figure, the maximum RCS value is obtained for an incident angle of 89.497°, which corresponds to the perpendicular to the leaning surface of the truncated cone. The oscillations shown are the PO sidelobes due to the currents induced on the surface of revolution. With the exception of oscillations, the results from (1) match simulation results. As it can be observed in Figure 9, the results provided by the proposed model have good agreement with the more computationally complex facets-based software tools.

## 7. Near Field Effects in the Scattering Analysis

The RCS formula assumes that far field condition in the scattering phenomenon is fulfilled, which implies that the object is illuminated by a plane wave. In practice, the range between the wind turbines and the radar is often smaller than the far field distance. In such a situation, the incident wave is spherical, and, therefore, the phase values of the incident wave at the surface of the body differ from the phase value at the center of the object. A widely accepted far field criterion is to limit the phase deviation in the radar receiver to be less than *π*/4, which means a maximum phase deviation of 22.5° in the incident wave on the object surface [39]. This condition is used to obtain the threshold distance for far field condition (*R*_0_) as a function of the lateral larger dimension of the object (*L*) and the wavelength *λ* [40–42]:

(4) $$\begin{array}{c} R_{0}=\frac{2L^{2}}{\lambda }. \end{array}$$

The near field effect, in the context of signal scattering and for monostatic reception, results in a reduction in the amplitude of the main lobe of the scattering pattern [39]. This effect can be comparable to considering an equivalent sized object instead of its actual size [41–43]. This equivalent size or “length in near field conditions” (*L*_nf_) is calculated according to the following expression:

(5) $$\begin{array}{c} L_{\text{nf}}=\sqrt{\frac{\lambda R}{2}}, \end{array}$$

where *R* is the distance from the radar to the object and *λ* is the radar wavelength. The length *L*_nf_ takes into account the cases where the far field condition is not fulfilled and provides a method to obtain the RCS of the object.

Additionally, a reduction of the directivity of the scattering pattern is also observed under near field conditions [15, 44, 45]. This implies that the main lobe of the scattering pattern is wider in the near field, and it supports the option of using the main value of the RCS as a representative value for the narrow set of angles related to marine radars.

In order to apply the near field effects, (1) must be expressed as a function of the largest dimension of the object (*L*). Consider

(6) σθn=8·π9·λ·(r23/2−r13/2)2(r2−r1)2 ·L2−(r2−r1)2{cos⁡⁡[atan⁡((r2−r1)×(L2−(r2−r1)2)−1)]}3.

The height of the mast must be replaced by the *L*_nf_  in near field condition:

(7) σθn=8·π9·λ·(r23/2−r13/2)2(r2−r1)2 ·Lnf2−(r2−r1)2{cos⁡⁡[atan⁡((r2−r1)×(Lnf2−(r2−r1)2)−1)]}3.

To verify the suitability of the above equation, the results are compared to the theoretical expression obtained by Welsh for the RCS of an upright cylinder in the near field condition when it is perpendicularly illuminated [39]. The ratio *σ*_meas⁡_/*σ*_ff_ (RCS in the near field with respect to RCS in the far field) obtained by Welsh is expressed as follows:

(8) $$\begin{array}{c} \frac{\sigma_{\mathrm{meas}}}{\sigma_{\text{ff}}}=\frac{\lambda R}{L^{2}}\left(C^{2}\left(\frac{L}{\sqrt{\lambda R}}\right)+S^{2}\left(\frac{L}{\sqrt{\lambda R}}\right)\right), \end{array}$$

where *C* and *S* are the cosine and sine Fresnel integrals.

**Figure 10:**
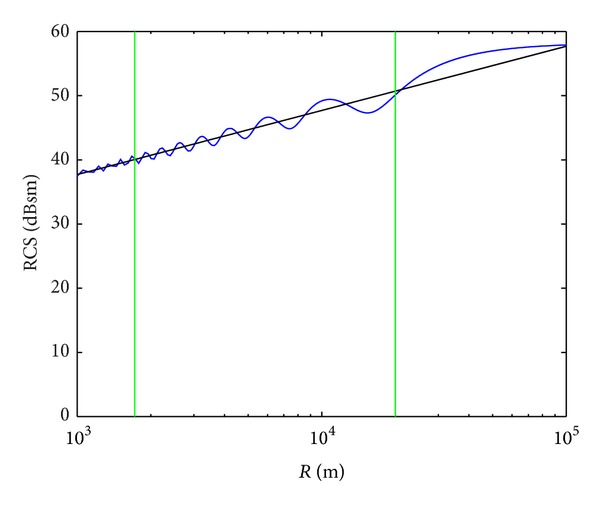
RCS values of a tapered cylinder, as a function of the distance to the radar. Comparison of the proposed method (black line) and Welsh's theoretical expression (blue line) [39]. Green lines delimit usual distances for offshore wind farms from the coastline [35].

The comparison for model 1 at 3.08 GHz is shown in Figure 10. As it can be clearly observed, the proposed method is in very good agreement with the theoretical results of Welsh, as the maximum difference is 2.3 dB. For the other turbine models and radar frequencies, the comparison provides differences less than 1.6 dB, which validates the characterization of the near field condition of the proposed model.

## 8. Verification of the Simplified Model against Other Models Implemented in PO-Based Tools

Although partial verifications of the proposed model have been already developed and described, in this section an overall evaluation of the proposed model is carried out by comparing the results with those provided by a different facets-based software tool for RCS estimation.

**Figure 11:**
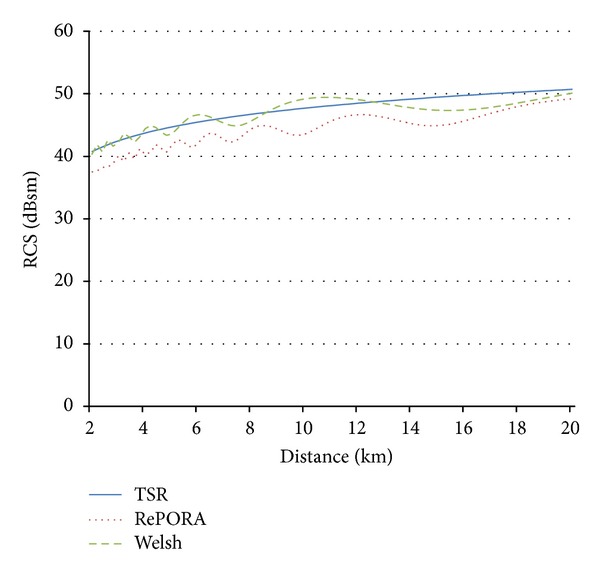
Comparison of the proposed method (in blue), RePORA model (red dotted line), and Welsh's theoretical expression (green dotted line) in near field for an incidence *θ*_*n*_ at 3.08 GHz for turbine model 1.

For this purpose, the RePORA model and Welsh's expression are used to assess RCS values as a function of the distance to the radar. In order to properly compare the maximum RCS values of the mast as a function of the distance in all the models, an upright cylinder is used to represent the turbine tower in RePORA model and Welsh's expression. The plots in Figure 11 show good agreement between both models.

**Figure 12:**
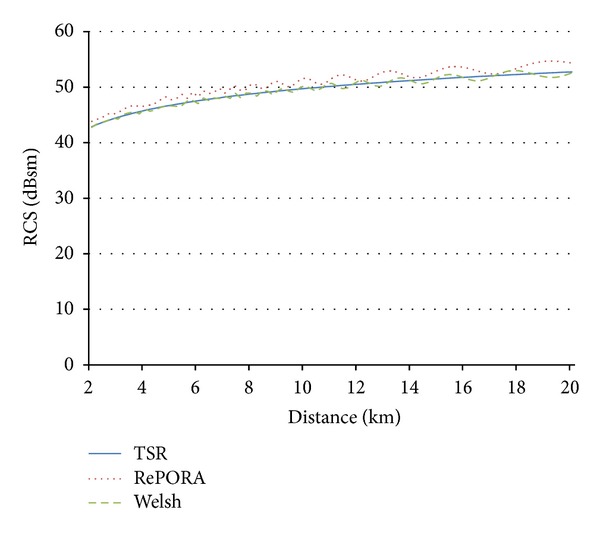
Comparison of the proposed method (in blue), RePORA model (red dotted line), and Welsh's theoretical expression (green dotted line) in near field for an incidence *θ*_*n*_ at 9.945 GHz for turbine model 3.

Figures 11 and 12 show the validity of the TSR simplified model for distances where offshore wind farms are usually installed (always near field conditions), as results of this model match the Welsh expression and results provided by RePORA model. The calculation time for obtaining representative RCS results is considerably lower, as the proposed formulae do not require the detailed representation of the wind turbine, neither the estimation of RCS values for different cases of rotor orientation and blades position.

## 9. Implementation of the Proposed Scattering Model

The proposed scattering model can be easily implemented in a software tool that automates the calculations of the false targets and clutter caused by a wind farm. Hence, the analysis can include real technical features of the signal transmitted by the radar and expected locations for wind turbines, in order to evaluate the potential impact that the wind farm may cause on the radar performance.

**Figure 13:**
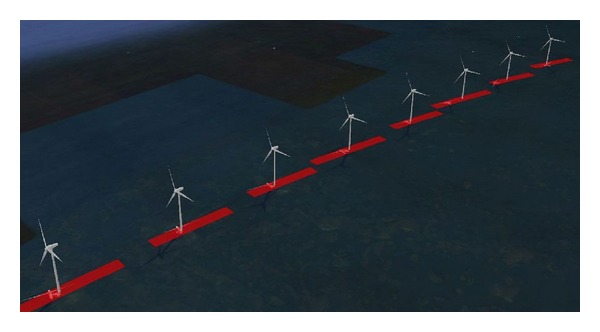
Assessment of the potential impact of Middelgrunden offshore wind farm (Denmark) on fictitious shore-based VTS radar. Red areas over the sea surface represent the clutter that the turbines would generate on the radar display [24, 25].

Figure 13 shows an example of the implementation of the proposed scattering model in a software tool [24, 25]. The potential impact of Middelgrunden offshore wind farm in Denmark on fictitious shore-based VTS radar has been assessed. Results of the calculations are shown in the figure, where a 3D setting of the wind farm is provided, and red areas over the sea surface represent the clutter that the turbines would generate on the radar display.

As illustrated, the proposed model greatly reduces the computational complexity and the required runtime and it does not involve complicated facetsbased wind turbine representations.

## 10. Conclusions

The disturbance that offshore wind turbines may cause in the performance of marine radars requires the characterization of wind turbine backscattering, in order to assess the potential impact before the wind farm is installed.

A computationally simple model for RCS estimation is proposed in this paper. The model provides a first approximation of the clutter level received by the radar antenna, in order to determine whether a new wind farm may degrade the detection capability of the surrounding marine radars. The method may be easily integrated into software tools to assess the impact of a specific wind farm in a real scenario.

Although sophisticated software tools for RCS estimation are currently available, they require detailed facets-based turbine representations, and they provide accurate results for a specific blades position. As the rotor orientation and blades rotation speed change continuously with the wind conditions, these complex calculations may require many runs over different orientation in order to give an average RCS value for general conditions.

For the case of marine radars and offshore wind turbines, the incidence angle is normal to the mast surface. In this situation, the mast is the main reflector of the turbine, and the scattering model may be based on this element. The monostatic RCS pattern for the mast has symmetry with respect to the vertical axis of the mast and a significant directivity in the vertical plane. Although this approach does not account for the RCS variation due to the rotation of the blades, this is deemed acceptable for impact assessment on marine navigational radars. The near field effects are included in the model as an equivalent size of the mast (*L*_nf_) in the formulation.

The proposed model has been validated for several important cases by means of facets-based software tools and empirical results. The proposed model may be easily implemented in a software tool for the analysis of the impact of real wind farms, and it will greatly reduce the computational complexity and the runtime required.

## Figures and Tables

**Table 1:** Offshore wind turbine models used in the analysis.

Dimensions		Turbine model 1	Turbine model 2	Turbine model 3
Mast	Height (m)	74	94	120
	Upper and lower diameters (m)	3.1–4.4	4–5.6	4.8–7.2
Rotor	Diameter (m)	111	128	126
	Blade length (m)	54	62.5	62
